# Construction of a Risk Model for Colon Cancer Prognosis Based on Ubiquitin-Related Genes

**DOI:** 10.5152/tjg.2023.22465

**Published:** 2023-05-01

**Authors:** Biwen Hu, Zhenwei Chen, Fei Yao, Buzhuo Li

**Affiliations:** 1 Department of General Surgery, The Second Affiliated Hospital of Jiaxing University, Jiaxing, China

**Keywords:** Bioinformatics, biomarkers, colon cancer, prognostic model, ubiquitination

## Abstract

**Background:**

Colon cancer is a frequently developed malignancy from the digestive system that leads to poor prognosis of patients due to its high recurrence and high metastasis. Dysregulation of ubiquitin-mediated signaling can result in tumor formation and metastasis. We aimed to develop prognostic markers related to ubiquitination in colon cancer and a risk assessment model based on these markers to improve the prognosis of colon cancer patients.

**Methods:**

We constructed a prognosis-related model by performing differential expression analysis on ubiquitin-related genes in colon cancer patients based on public data and then undertaking Cox analysis, which selected 7 ubiquitin-related prognostic genes (TRIM58, ZBTB7C, TINCR, NEBL, WDR72, KCTD9, and KLHL35). The samples were divided into high and low RiskScore groups according to the risk assessment model, and as Kaplan–Meier suggested, the overall survival of patients with high RiskScore was prominently lower than that of patients with low RiskScore. The accuracy of RiskScore was assessed by receiver operating characteristic curves. Accordingly, the area under the curve values of 1-, 3-, and 5-year were 0.76, 0.74, and 0.77 in the training set and 0.67, 0.66, and 0.74 in the validation set, respectively.

**Results:**

These data confirmed the preferable performance of this prognostic model in predicting colon cancer patients’ prognoses. The relationship between this RiskScore and clinicopathological factors of colon cancer patients was analyzed via stratification. Univariate and multivariate Cox regression analyses were performed to determine whether this RiskScore could be applied as an independent prognostic factor. Finally, in order to better apply the prognostic model in clinical practice, we constructed an overall survival nomogram for colon cancer patients’ prognoses based on clinical factors and RiskScores, which has preferable prediction accuracy and is better than the traditional tumor, node, and metastasis (TNM) staging system.

**Conclusions:**

The overall survival nomogram for prognosis can assist clinical oncologists to make a more accurate evaluation of patients’ prognosis, as well as the implementation of individualized diagnosis and treatment for colon cancer patients.

**Main Points**

- In this study, differentially expressed ubiquitin-related genes in colon cancer (CC) were identified.
- A 7-gene model was constructed for prognostic risk assessment in CC.
- This study screened for the prognostic markers on the basis of ubiquitin-related genes in CC patients.

## INTRODUCTION

Cancer is threatening global public health with about 19.3 million new cases in 2020, among which colorectal cancer ranks third for 10% of incidence and second for 9.4% of mortality.^1^ Colon cancer (CC) is one of the most commonly developed malignancies of the digestive tract, which can be regarded as a marker of socioeconomic development. Its incidence often rises stably with the human development index in countries undergoing a major transformation.^2^ Besides, factors such as aging population, diet, obesity, lack of exercise, as well as smoking also increase the risk of colorectal cancer.^3^ Despite the rapid development of early diagnosis and treatment of CC over the past decades, the prognosis of patients remains deadly poor due to its high recurrence and metastasis.^4-6^ Therefore, the dissection of tumorigenesis and prognostic markers in CC is an important way of preventing and controlling CC.

Tumor development depends on dysregulated genes and post-translational regulation, and as one of the main pathways of protein post-translational modification, ubiquitination is ubiquitous in organisms, whose process is able to regulate a variety of protein substrates existing in different cellular pathways.^7,8^ The ubiquitin linkage of proteins is catalyzed by E3 ubiquitin ligase and ATP via a 3-enzyme cascade (E1–E2–E3) to bind to the degraded substrate protein, which is degraded and cleared by mediating proteasome.^9^ Ubiquitination means a lot to the regulation of the synthesis and catabolic processes of proteins in organisms, and a recent report revealed that ubiquitination can also be involved in regulating various cell biological events such as gene transcription, cell cycle progression, DNA damage repair, and apoptosis.^10^ As reported, ubiquitination can modulate pathways that control either tumor suppression or promotion.^11,12^ At present, bunches of studies have targeted the ubiquitin-related genes in tumors to construct models. To take an example, Cai et al^13^ constructed a 6-gene prognostic model for bladder cancer on the basis of ubiquitin-related genes. Che et al^14^ combined ubiquitination with immunization to screen 26 ubiquitinated genes associated with the prognosis of patients with lung adenocarcinoma (LUAD) and constructed 3 prognostic risk assessment models of LUAD via bioinformatics approaches. Given that, it is reasonable to predict the prognosis of CC patients by identifying ubiquitination-related genes, which will add to the basis for individualized treatment.

In our research on CC, we utilized a dataset from public databases to develop and validate a prognostic signature for CC based on ubiquitin-related genes. Besides, we performed a comprehensive analysis of the feature genes to improve the clinical utility of these markers. In conclusion, the signature we constructed can effectively predict the prognosis of CC patients and has the potential for clinical applications.

## Materials and Methods

### Data Source

We downloaded mRNA expression data (normal: 41, tumor: 473) of CC patients from The Cancer Genome Atlas (TCGA-COAD) (https://portal.gdc.cancer.gov/) dataset in the count format and collected their clinical data. Subsequently, we adopted the iUUCD 2.0 database (http://iuucd.biocuckoo.org/index.php) to collect ubiquitin-related human genes, including 9 E1 (ubiquitin-activating enzyme), 43 E2 (ubiquitin-conjugating enzyme), 919 E3 (ubiquitin ligase), 126 DUBs (deubiquitinating enzyme), 387 UBD (proteins containing ubiquitin-binding domains), and 113 ULD (proteins containing ubiquitin-like domains) (https://portal.gdc.cancer.gov/).

### Selection of Ubiquitin-Related Genes in Colon Cancer

Differential expression analysis was performed on mRNA expression data in TCGA-COAD dataset by using the R package “edgeR”^15^ to identify differentially expressed genes (DEGs) in CC. These DEGs were then intersected with ubiquitin-related genes to yield ubiquitin-related genes that were differentially expressed in CC for subsequent analyses.

### Screening of Prognosis-Related Ubiquitin-Related Genes and Construction of a Prognostic Risk Assessment Model

Based on the survival time of patients in the dataset, we selected data with patients’ survival time greater than 0. The screened data set was randomly divided into the train set (n = 299) and the validation set (n= 127) at a ratio of 7:3. Ubiquitin-related genes data in the validation set were subject to univariate Cox regression analysis by R package “survival”^16^ (*P* < .05) (https://cran.r-project.org/web/packages/survival/index.html). To prevent the model from overfitting, lasso regression analysis was also performed on these genes using the R package “glmnet,”^17^ and by using the cross-validation method, the penalty function lambda was our tool for removing the overfitting genes to reduce the fitting degree of the model. Finally, multivariate Cox regression analysis was performed on the obtained genes using the R package survival to construct a prognostic risk assessment model for ubiquitin-related genes in CC. The formula for RiskScore was:


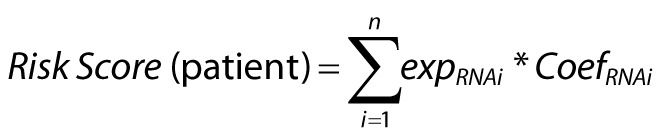



where Coef value is the regression coefficient obtained by multivariate Cox regression analysis, and ubiquitin-related RNA expression of patients is defined as exp_RNA_.

### Evaluation of the Performance of the Model

Based on the expression level and risk coefficient of samples’ feature genes screened by the model, we calculated the RiskScore of each sample in the train set. Samples larger and less than the median RiskScore were divided into high- and low-risk groups, respectively. Thereafter, we drew the survival curves and receiver operating characteristic (ROC) curves of both groups using the R package “survival” and “timeROC”^18^ to calculate the area under the ROC curve (AUC) of 1-, 3-, and 5-year overall survival (OS). Samples in the validation set were used for validation, so as to evaluate the predictive ability of the prognostic risk assessment model constructed in this study.

### Evaluation of the Independence of the Model

To assess whether RiskScore could serve as an independent prognostic factor, univariate and multivariate Cox regression analyses were performed in combination with age, gender, grade, tumor, node stage (TN stage), as well as RiskScore. The clinical factors and RiskScore together generated a nomogram by the R package “rms,”^19^ so as to predict the OS of patients at 1, 3, and 5 years. Subsequently, the calibration curve of the nomogram was generated using the R package “foreign” to verify the prediction performance of the nomogram.

## RESULTS

### Selection of Differentially Expressed Ubiquitin-Related Genes in Colon Cancer

**Figure 1.**
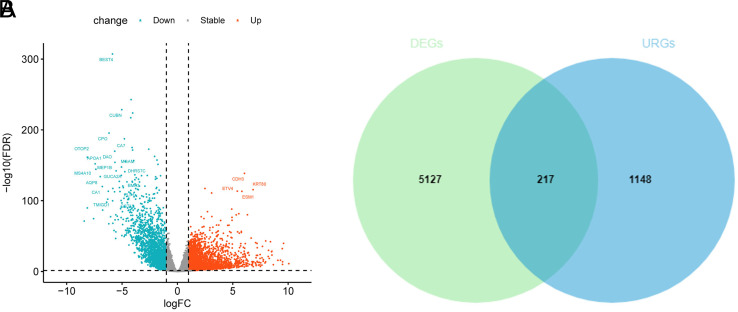
Screening of ubiquitin-related genes in CC. (A) Volcano plot of differential expression analysis in CC (blue for downregulated ones and red for upregulated ones). (B) Screening of ubiquitin-related genes (green for DEGs in CC and blue for ubiquitin-related genes in the database). CC, colon cancer; DEGs, differentially expressed genes.

First, 5344 DEGs were yielded by differential expression analysis on the data from the TCGA-COAD dataset using the R package, including 3015 upregulated ones and 2329 downregulated ones (Figure 1A). Subsequently, ubiquitin-related genes of tumors were downloaded from the iUUCD 2.0 database, while unmatched gene symbols and repeated genes were removed. And DEGs were intersected with the rest of ubiquitin-related genes, by which 217 ubiquitin-related genes were finally acquired (Figure 1B).

### Screening of Ubiquitin-Related Characteristic Genes and Construction of the Prognostic Model

**Figure 2.**
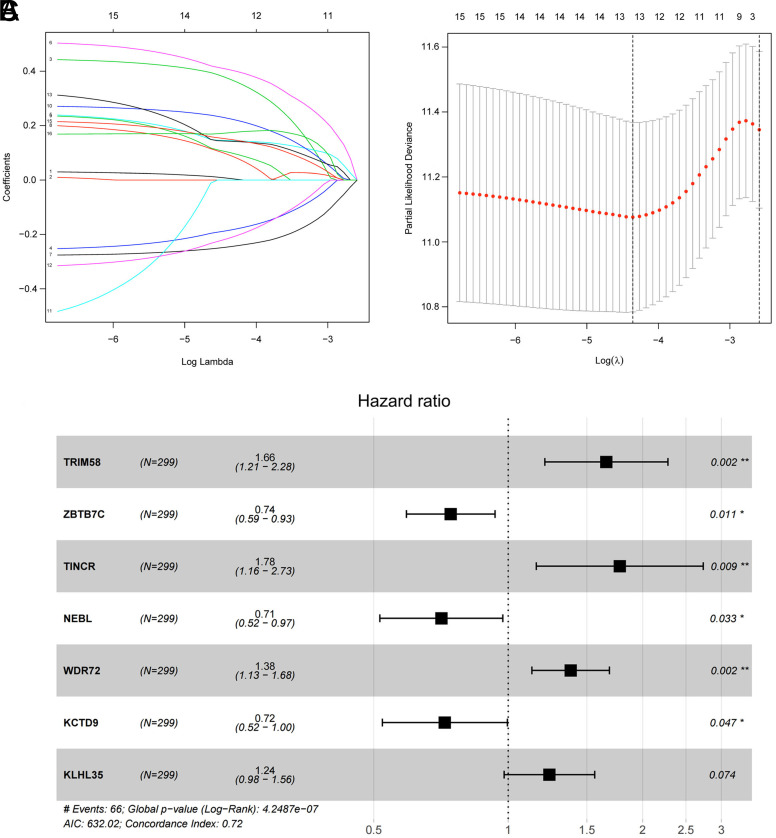
Screening of ubiquitin-related characteristic genes and construction of prognostic model. (A, B) LASSO analysis of 16 prognosis-related genes. (C) Forest plot of the ubiquitin-related 7-gene prognostic model.

First, 16 ubiquitin-related genes related to CC patients’ OS were initially screened via univariate Cox analysis (Supplementary Table 1). To prevent overfitting, the results of univariate Cox analysis were further subject to Lasso regression analysis, which yielded 13 important ubiquitin-related genes (Figure 2A and 2B). Further, a multivariate Cox analysis revealed that 7 optimal ubiquitin-related genes were prognosis-related (TRIM58, ZBTB7C, TINCR, NEBL, WDR72, KCTD9, and KLHL35) (Figure 2C), of which ZBTB7C, NEBL, and KCTD9 were protective factors, and TRIM58, TINCR, WDR72, and KLHL35 were risk factors, and the risk model was: RiskScore = 0.5060 * TRIM58 − 0.2963 * ZBTB7C + 0.5751 * TINCR − 0.3448 * NEBL + 0.3218 * WDR72 − 0.3266 * KCTD9 + 0.212435 * KLHL35.

### Analysis of the Prognostic Model

**Figure 3.**
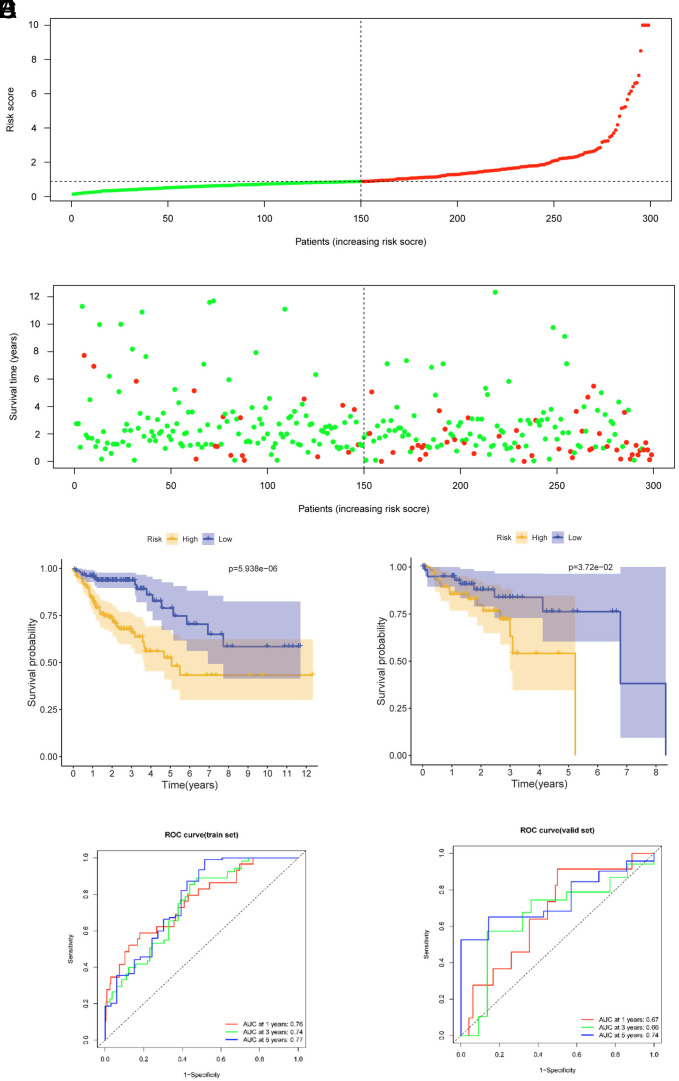
Screening of ubiquitin-related prognostic genes in CC and construction of the prognostic model. (A) Distribution plot of RiskScores for CC patients (green for low-risk group and red for high-risk group). (B) Scatter plot of survival status of CC patients (green for low-risk group and red for high-risk group). (C, D) Kaplan–Meier survival curves of CC patients in different risk groups in the training set and the validation set (blue for low-risk group and yellow for high-risk group). (E, F) ROC curves of the 7-gene risk model in the training set and the validation set. CC, colon cancer; ROC, receiver operating characteristic.

We input patients’ RNA expression into the prognostic model to calculate the RiskScore and divided the patients into different risk groups according to the median value of the RiskScore. Meanwhile, the survival distribution map and survival curves of CC patients were plotted and validated using the validation set. As the results indicated, the survival time of patients was shortened with the RiskScore increased (Figure 3A–D). To validate the accuracy of the model, we plotted ROC curves and assessed the performance of the model on the basis of the AUC. As indicated by RiskScore, the AUC values of 1-, 3-, and 5-year survival were 0.76, 0.74, and 0.77 in the training set, respectively, and 0.67, 0.66, and 0.74 in the validation set, respectively (Figure 3E and 3F). Apparently, the prognosis of CC patients could somewhat be predicted by the ubiquitin-related gene model we constructed based on the data from TCGA-COAD dataset.

### Validation of Dependency and Clinical Significance of the Model

**Figure 4.**
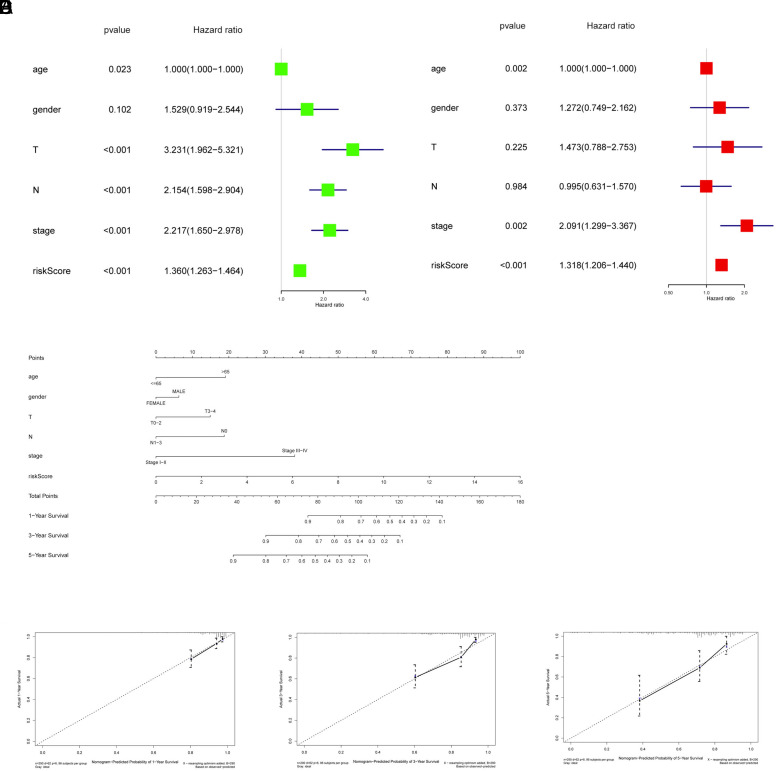
Application of the risk assessment model to clinical factors. (A) Forest plot of univariate Cox regression of 7-gene RiskScore and clinical factors. (B) Forest plot of multivariate Cox regression of 7-gene RiskScore and clinical information. (C) Nomogram of 7-gene RiskScore and clinical information. (D–F) Calibration curve of the nomogram in predicting 1-, 3-, and 5-year survival of CC patients. CC, colon cancer.

We here explored via Cox analysis whether the 7-gene prognostic model we constructed was qualified to be an independent prognostic factor by combining traditional clinical factors (age, gender, T, N, stage) and the RiskScore. Univariate Cox analysis indicated that significance lay in RiskScore, T, N, and stage (Figure 4A), while multivariate analysis indicated that significance lay in RiskScore and age (Figure 4B). Combining COX analysis results, the RiskScore generated from the prognostic risk model constructed based on CC patients’ ubiquitin-related genes had the ability to be an independent prognostic factor. To better apply the model to the clinic practice, we constructed a nomogram by combining RiskScore with 5 clinical factors (Figure 4C). To assess the accuracy of the nomogram, we also plotted the 1-, 3-, and 5-year calibration curves of the nomogram, which indicated overfitting of the characteristic situation, suggesting that our nomogram constructed according to the RiskScore and clinical factors could well predict the survival of patients (Figure 4D–F). In sum, the above results suggested that the risk assessment model constructed herein could effectively predict the prognosis of CC patients.

## DISCUSSION

Current studies on cancer biology revealed that the ubiquitination pathway of protein post-translational modification has a key role in regulating cellular processes. To take an example, the ubiquitination process can maintain cellular homeostasis in substrate degradation by regulating the quantity and quality of proteins. At the same time, the ubiquitin-protease system is fundamental in maintaining cellular metabolism, viability, and cell cycle regulation, and its dysregulation can cause cancer development.^20^ There is research on ubiquitin-related genes in CC. Yue et al^21^ reported that upregulated miR-340-5p or downregulated ATF1 can impact on the ubiquitination of FOXA1 by affecting the E3 ubiquitin ligase NEDD4 level, thereby promoting the malignant CC progression. Yu et al^22^ also reported that USP47 and SMURF2 can mediate CC cell proliferation and tumor progression by reversibly manipulating SATB1 ubiquitination. Accordingly, it is pivotal to mine ubiquitin-related genes in patients, so as to explore tumor pathogenesis.

We screened 7 feature genes (TRIM58, ZBTB7C, TINCR, NEBL, WDR72, KCTD9, and KLHL35) by differentially analyzing CC patients’ ubiquitin-related genes and thereby constructed a risk assessment model for CC prognosis. Three of these genes were protective genes, which were ZBTB7C, NEBL, and KCTD9. As a member of ZBTB family, ZBTB7C has a BTB structure at the N-terminal and multiple zinc fingers at the C-terminal.^23^ ZBTB7C is a candidate tumor suppressor gene that is lowly expressed or even silenced in most cervical cancer cell lines but expresses in normal cervical epithelial cells.^23^ Meanwhile, Chen et al^24^ exhibited that ZBTB7C could affect CC cells via different pathways or by targeting miRNAs, and ZBTB7C is lowly expressed in CC, which is linked to poor prognosis of CC patients. Our findings were consistent with this research. NEBL is a crucial protein for cell adhesion and actin filament structure in cells, which manipulates the migration of cytoplasmic matrix attachment and is abundantly expressed in cardiomyocytes.^25^ As reported, NEBL performs either as an oncogene or as a tumor suppressor in cancer. To take an example, Wang et al^26^ reported that NEBL is downregulated in colorectal cancer and represses the malignant progression of colorectal cancer. KCTD9 belongs to the KCTD family that contains a unique specific DUF3354 domain and pentapeptide repeats in eukaryotes. Although few studies focus on KCTD9, which is mainly related to viral hepatitis and liver failure, no study revealed its effect on tumors. However, the existing reports revealed that KCTD9 will affect the biological functions of NK cells. Zhang et al^27^ confirmed that NK cells containing silenced KCDT9 will weaken cell cytotoxicity of tumors in vitro. Although there is no study to verify the effect of KCTD9 on tumor cells, it can be seen that KCTD9 will affect innate immune cells in humans. In combination with our work, KCTD9 level was higher in patients with low RiskScore over those with high RiskScore. Accordingly, KCTD9 may affect the malignant progression of tumors via the innate immunity of tumors.

At the same time, there were 4 risk genes in this risk assessment model, which were TRIM58, TINCR, WDR72, and KLHL35. TRIM58 is an E3 ubiquitin ligase^28^ that has been demonstrated to be a potential prognostic marker for colorectal cancer, which can repress cancer cell invasion by initiating EMT and MMP.^29^ TINCR is the lncRNA that is most upregulated during cell differentiation. TINCR can interact with copious mRNAs and proteins to manipulate mRNA differentiation.^30,31^ Previous studies implied that TINCR is closely related to the proliferation and apoptosis of cells in various tumors, including breast cancer, gastric cancer, and hepatocellular carcinoma.^30,32,33^ Zhang et al^34^ and Ren et al^35^ proved that overexpressed TINCR could repress the proliferation of colorectal cancer cells and facilitate apoptosis. WDR72 is a protein with functions unknown and is less studied in tumors. Nevertheless, a bunch of studies has indicated that it serves as a biomarker for plenty of cancers, like renal cell carcinoma, esophageal cancer, as well as triple-negative breast cancer.^36,37^ The specific function of KLHL35 is also elusive, but as exhibited in studies, KLHL35 is associated with DNA methylation and tumor mutation burden in cancers like aortic aneurysm, LUAD, and renal cell carcinoma.^38-40^ To conclude, this research screened prognostic markers on the basis of ubiquitin-related genes in CC patients, and these markers indicated a high value for predicting the prognostic risk in clinical practice and is expected to be a therapeutic target as well as a biomarker for CC.

Nevertheless, there were still some limitations in this study. This study yielded a prognostic risk assessment model on the basis of a public database, which was not been validated by prospective clinical experiments. Besides, basic experiments such as in vitro cell experiments, and in vivo animal experiments are needed to dissect the potential mechanism of these ubiquitin-related genes affecting CC progression.

## Figures and Tables

**Supplementary Table 1.** Results of Univariate Cox Analysis

DYNC1I1	KLHL17	TRIM58	ZBTB7C	RNF208	TINCR	NEBL	PACSIN1	UBXN11	WDR72	ANKRD13D	KCTD9	BTBD19	UCHL1	KLHL35	DCAF4L2
